# Insight into the evolution of the proton concentration during autohydrolysis and dilute-acid hydrolysis of hemicellulose

**DOI:** 10.1186/s13068-016-0619-6

**Published:** 2016-10-21

**Authors:** Nuwan Sella Kapu, Zhaoyang Yuan, Xue Feng Chang, Rodger Beatson, D. Mark Martinez, Heather L. Trajano

**Affiliations:** 1 Department of Chemical and Biological Engineering, University of British Columbia, 2360 East Mall, Vancouver, V6T 1Z3 Canada; 2 Chemical and Environmental Technology, British Columbia Institute of Technology, 3700 Willingdon Ave, Burnaby, V5G 3H2 Canada; 3 Department of Wood Science, University of British Columbia, 2424 Main Mall, Vancouver, V6T 1Z4 Canada

**Keywords:** Autohydrolysis, Dilute-acid hydrolysis, Biomass, Pretreatment, Kinetic modeling, Hemicellulose, Proton concentration

## Abstract

**Background:**

During pretreatment, hemicellulose is removed from biomass via proton-catalyzed hydrolysis to produce soluble poly- and mono-saccharides. Many kinetic models have been proposed but the dependence of rate on proton concentration is not well-defined; autohydrolysis and dilute-acid hydrolysis models apply very different treatments despite having similar chemistries. In this work, evolution of proton concentration is examined during both autohydrolysis and dilute-acid hydrolysis of hemicellulose from green bamboo. An approximate mathematical model, or “toy model”, to describe proton concentration based upon conservation of mass and charge during deacetylation and ash neutralization coupled with a number of competing equilibria, was derived. The model was qualitatively compared to experiments where pH was measured as a function of time, temperature, and initial acid level. Proton evolution was also examined at room temperature to decouple the effect of ash neutralization from deacetylation.

**Results:**

The toy model predicts the existence of a steady-state proton concentration dictated by equilibrium constants, initial acetyl groups, and initial added acid. At room temperature, it was found that pH remains essentially constant both at low initial pH and autohydrolysis conditions. Acid is likely in excess of the neutralization potential of the ash, in the former case, and the kinetics of neutralization become exceedingly small in the latter case due to the low proton concentration. Finally, when the hydrolysis reaction proceeded at elevated temperatures, one case of non-monotonic behavior in which the pH initially increased, and then decreased at longer times, was found. This is likely due to the difference in rates between neutralization and deacetylation.

**Conclusions:**

The model and experimental work demonstrate that the evolution of proton concentration during hydrolysis follows complex behavior that depends upon the acetyl group and ash content of biomass, initial acid levels and temperature. In the limit of excess added acid, pH varies very weakly with time. Below this limit, complex schemes are found primarily related to the selectivity of deacetylation in comparison to neutralization. These findings indicate that a more rigorous approach to models of hemicellulose hydrolysis is needed. Improved models will lead to more efficient acid utilization and facilitate process scale-up.

## Background

In this work, the kinetics of proton generation during prehydrolysis of bamboo chips in a batch reactor is examined. Bamboo grows rapidly to become harvest-ready in approximately three years, and has a chemical composition similar to wood [1, 2]. It is also an abundant natural resource in many Asian countries. For example, China is reported to be the home for over 500 species of bamboo covering more than seven million hectares [3]. Moreover, bamboo is considered a promising species for cultivation on marginal land for biofuels and bio-products [3]. Despite these advantages, it is only recently that bamboo has garnered research focus in the area of pulping and biorefinery applications [1, 4], and it can still be considered an underutilized feedstock.

Prehydrolysis, which is also referred to as ’pretreatment’, refers to the reaction pathway to remove hemicelluloses from lignocellulosic material during the production of high-purity dissolving pulps or biofuels [3, 5, 6]. Here, an acid catalyzes the breakdown of long hemicellulose chains to form shorter chain oligomers and sugar monomers in the presence of water or steam. Kinetic modeling still remains at the forefront and the evolution of concentration of the acid catalyst ${[\mathrm{H}^+]}$ is one of the longstanding unanswered questions [7]. Prehydrolysis is different from torrefaction wherein biomass is treated at 200–300 °C in an inert gas environment [8]. Hemicellulose is hydrolyzed into mostly soluble sugars during prehydrolysis, while during torrefaction, it is degraded, depending on process temperature, into volatile organic compounds including CO_2_ and CO, and char [9]. Prehydrolysis is typically performed by treating biomass at 140–180 °C with either water/steam (autohydrolysis) or dilute acid solutions [6]. Autohydrolysis is an industrially practiced step in dissolving pulp production, and both autohydrolysis and dilute-acid hydrolysis are considered viable pretreatment options in the production of lignocellulosic ethanol. However, the chemical complexity of biomass and the lack of refined kinetic models continue to hamper process optimization and scale-up efforts.

The literature on the kinetics of the removal of hemicellulose is substantial. The modeling approach was built upon the approach used for dilute-acid hydrolysis of cellulose [10]. For hemicellulose, complex behavior is evident and numerous groups consider that two fractions of hemicellulose are distributed spatially over two separate domains in the solid matrix to help simplify the analysis [11]. Each fraction reacts with the available protons at differing rates due to differences in reaction activation energy. This model has been adopted widely and is commonly referred to as the “biphasic model”. It consists of two solid species, fast and slow hemicelluloses, denoted as ${X_i}( {\rm{s}})$, which hydrolyze following first-order kinetics

1 $$\begin{aligned} {X}_{i}(\mathrm{s}) \xrightarrow [{\mathrm{H}^{+}}]{{\mathrm{k}_\mathrm{i}}} {X}(\mathrm{aq}) \qquad \qquad r_i = k_i[{X}_{i}], \end{aligned}$$

where $r_i$ and $k_i$ are defined as the rate of reaction and rate constant, respectively, to form a set of soluble products, *X*(aq), which are susceptible to further hydrolysis or decomposition reactions. The subscript *i* represents either fast or slow. The initial values for ${X}_{i}$ are considered to be intrinsic for the biomass [12, 13]. Variations on this approach are available in the literature to describe subtle effects such as the formation of oligomeric intermediates or mass transfer rates [1, 14–28]. However, no physical or chemical attributes have been identified to differentiate fast and slow hemicelluloses.

One of the open remaining questions in this literature is an understanding of the evolution of the concentration of the acid catalyst. What makes this problem particularly challenging is that there are competing pathways governing proton evolution and neutralization. Although difficult to substantiate, a number of authors have advanced rate constants $k_i$ of the form

2 $$\begin{aligned} k_i = {k_o}_i \exp \left(-\frac{{E_a}_i}{RT}\right)f\left(t,[\mathrm{H}^+]\right), \end{aligned}$$

where ${k_o}_i$ is the pre-exponential factor, and ${E_a}_i$ is the activation energy. The function $f(t,{[\mathrm{H}^+]})$ is determined empirically and is found to vary greatly in the literature. This function is included to allow for different reaction rates with different acid levels. In one extreme, we find that this function varies linearly in time while in the other extreme it is considered as a constant and set to its initial value. We summarize these forms as

3 $$\begin{aligned} f\left( t,\left[ {{ \mathrm H}^{+}} \right] \right) =\left\{ \begin{matrix} a+bt &{}&{} \text {autohydrolysis} \\ \left[ {\mathrm{H}^{+}} \right] _0^{n} &{}&{} \text {dilute acid} \\ \end{matrix} \right. \end{aligned}$$

depending upon if the experiment is conducted under dilute-acid or autohydrolysis conditions. Here, *a*, *b*, and *n* are empirical constants and ${[\mathrm{H}^{+}]^n_0}$ is the initial concentration of the acid catalyst. *n* is typically found to be between 0.8 and 1.3 and we note that Shen and Wyman [24] set $n=1$ for corn stover. The utility of this functional form has been questioned and it is evident that there is no theoretical basis for the form of the assumed functions [12, 15, 22, 29–35]. In this work, we attempt to gain insight into the assumed form of Eq. 3 by examining the evolution of the proton concentration during reaction through experiment and mathematical modeling.

## Model development

The analysis presented in this section is aimed at understanding the evolution of ${[\mathrm{H}^+]}$ during reaction. The goal is to develop a qualitative understanding of this form by posing a hypothetical reaction scheme which, at some level of approximation, represents the true reaction scheme. It is done at a level in which the analysis is mathematically transparent and of sufficient detail to capture the dominant mechanisms. As a result, we refer to our approach in the subsequent comparison to the experimental data as “the toy model”.

**Fig. 1:**
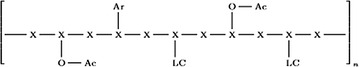
A schematic of the idealized hemicellulose (X)–lignocellulose (LC) substrate considered in this work. Although xylan is hypothesized to be comprised of fast and a slow-reacting fractions, we do not distinguish these in this figure. The species Ac and Ar, which represent the acetyl and arabinose groups, are initially bound to the xylan chain but are released through acid hydrolysis. The ash (MO) is not shown in this figure but is considered to be physically embedded in the LC portion of the matrix

One of the many complicating factors hindering the modeling process is that there is a large number of chemical species (Table 1 highlights this), which are distributed throughout the cell wall in a complex manner. To simplify, classes of species which behave similarly are grouped together and represented as one hypothetical species. For example, we represent the ash constituents as a lumped parameter MO, that is, the ash is an oxide of the species M with a valence state of $2^+$; this hypothetical species serves to neutralize the available protons. This can be reposed at another valence state or with secondary effects, such as precipitation from solution, included. In a similar manner the hemicellulose constituents have been reduced to a linear xylose polymer, denoted by *X*, fast and slow, having arabinose (Ar) side chains (Fig. 1). Protons are represented by ${\mathrm{H}}^+$ and the hydroxyl groups by ${\mathrm{OH}}^-$; both of these species are considered to be in the aqueous phase and the aq notation has been dropped. We have included the potential of an acid being added to the system and denote this species as ${\mathrm{H}_2\mathrm{A}}$ because sulfuric acid is most commonly used in the literature. The acetyl group Ac is defined as ${\mathrm{H}_{3}\mathrm{C}{-}\mathrm{C}{=}(\mathrm{O})^-}.$ Mass transfer effects are neglected.

**Table 1:** Representative composition of the bamboo chips

Composition	% od, bamboo
*Hemicellulose as*	24.1
Xylan	22.3
Arabinan	1.1
Galactan	0.7
*Ash as*	2.1
SiO_2_	1.1
CaO	0.4
K_2_O	0.3
Al_2_O_3_	0.2
*Cellulose as*	48.7
α	47.3
β	1.4
*Lignin as*	25.1
Acid soluble	0.9
Acid insoluble	24.2

**Fig. 2:**
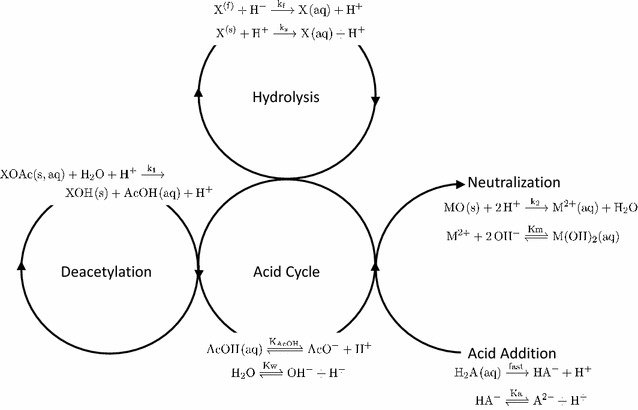
A schematic of the idealized reaction scheme. The chemical reactions shown form the basis of the toy mathematical model of the proton concentration

We consider four primary reactive pathways in Fig. 2 and each individual reaction is assumed to follow elementary kinetics. In the first of these, shown on the far left of Fig. 2, we consider deacetylation where Ac is cleaved from the hemicellulose backbone though an acid hydrolysis of the ester

4 $$\begin{aligned} \mathrm{XOAc} + {\mathrm{H}_{2}\mathrm{O}}\xrightarrow [{\mathrm{H}^{+}}]{{\mathrm{k}_{1}}} \mathrm{XOH}(\mathrm{s}) + \mathrm{AcOH}(\mathrm{aq}) \\ r_1 = k_1[\mathrm{XOAc}][\mathrm{H}^+]. \end{aligned}$$

This reaction may occur with acetyl groups which are attached to either soluble or solid phases of the hemicellulose. For simplicity any differences in rate between the deacetylation reaction occurring in the solid or liquid phases are ignored. As the product AcOH(aq), acetic acid, behaves as a weak acid, it adopts the following equilibrium in solution

5 $$\begin{aligned} \mathrm{AcOH}(\mathrm{aq}) & {\overset{{{K}_\mathrm{AcOH}}}{\rightleftharpoons }} \mathrm{AcO}^{-} + \mathrm{H}^{+},\\  K_{\mathrm{AcOH}} &= \frac{[\mathrm{AcO}^-][\mathrm{H}^{+}]}{[\mathrm{AcOH}]}= 1.8 \times 10^{-5}\, M, \end{aligned}$$

where $K_{i}$, from this point forward is defined as the equilibrium constant and the value quoted is at room temperature. Both Garrote et al. [18] and Aguilar et al. [26] have used similar modeling approaches to describe deacetylation. Aguilar et al. for example, explicitly indicated that this reaction follows first-order kinetics [26]. We build upon these studies by including the effects of the weak-acid behavior of acetic acid (see Eq. 5). Water disassociation

6 $$\begin{aligned} \mathrm{H}_{2}\mathrm{O} & {\overset{K_{\rm {w}}}{\rightleftharpoons }} \mathrm{OH}^{-} + \mathrm{H}^{+}\\{K_{\rm {w}}} &= [\mathrm{OH}^-][\mathrm{H}^+]=1\times 10^{-14}\,M^2 \end{aligned}$$

is an additional source of H^+^. Because of these equilibria, H^+^ is available for both the neutralization and hydrolysis reactions.

In addition to this, protons may also be available if acid is added to the system. We capture the reaction scheme as if the added acid is sulfuric acid, as this is the most common addition in the literature:

7 $$\begin{aligned}&{\mathrm H}_{2}{\mathrm A}({\mathrm {aq}}) \xrightarrow {\mathrm {fast}} {\mathrm {HA}}^{-} + {\mathrm H}^{+} \end{aligned}$$

8 $$\begin{aligned}{\mathrm {HA}}^{-} & {\overset{ K_{\mathrm{a}}}{\rightleftharpoons }} {\mathrm A}^{2-} + {\mathrm H}^{+}\\ & { K_{\rm{a}}} = \frac{{[{\mathrm {A}}^{2-}][{\mathrm {H}}^{+}]}}{{[{\mathrm {HA}}^{-}]}}= 1\times 10^{-2}\,M \end{aligned}$$

Like others in the literature, we consider the disassociation given in Eq. 7 to be instantaneous. The final aspect to consider is the neutralization of the protons by the ash. As mentioned above the reaction scheme depends upon the species involved. Here, we considered a hypothetical oxide MO which reacts according to the following scheme

9 $$\begin{aligned} \mathrm{MO}(\mathrm{s}) + \mathrm{2H}^{+} \xrightarrow {k_2} \mathrm{M}^{2+}(\mathrm{aq}) + \mathrm{H}_2\mathrm{O} \\ r_2= & {} k_2 [\mathrm{MO}(\mathrm{s})][\mathrm{H}^+] \end{aligned}$$

10 $$\begin{aligned}\mathrm{M}^{2+} + 2\mathrm{OH}^- &{\overset{K_{\mathrm {m}}}{\rightleftharpoons }} \mathrm{M}(\mathrm{OH})_2(\mathrm{aq})\\{K_{\rm {m}}}&=  \frac{[\mathrm{M}^{2+}(\mathrm{aq})][\mathrm{OH}^-]^2}{[\mathrm{M(OH)}_2]} \rightarrow 0 \end{aligned}$$

As the equilibrium constant ${K}_\mathrm{m}$ is unknown, we simply assign this value to be a very small number to reduce the number of free parameters. It should be noted that we do not characterize a number of the potential secondary reactions in solutions, even though they may affect the proton levels to a small degree. For example, we ignore the potential reaction between ${M^{2+}}$ and ${A^{2-}}$ for mathematical transparency as these do not effect the proton concentration.

Having established the chemistry of the toy model, we now construct the mathematical model. We build the model upon two conservation laws: conservation of mass of each of the species found in solution and an overall charge neutrality of the solution. Conservation of mass expresses that the initial moles of a certain species must sum to the total moles of the species in the reaction products. For example, the initial moles of M in ${[\mathrm{{MO}}]_{0}}$ must balance the number of moles of M, in the species [MO], ${[\mathrm{M}^{2+}]}$, and ${[\mathrm{{M(OH)}}_2]}$ at any time throughout the course of the reaction. This can be expressed as

11 $$\begin{aligned}{}[\mathrm{MO}]_0&= [\mathrm{MO}]+ [\mathrm{M}^{2+}] + [\mathrm{M(OH)}_2] = [\mathrm{MO}]+ [\mathrm{M}^{2+}]\left( 1 + \frac{[{ \rm {OH}}^-]^2}{{ K}_{\rm {m}}}\right) \end{aligned}$$

through use of the equilibrium relationship given in Eq. (10). In a similar manner, conservation of mass for the species Ac can be expressed as

12 $$\begin{aligned}{}[\mathrm{XOAc}]_o &= [\mathrm{XOAc}]+ [\mathrm{AcOH}]&+ [\mathrm{AcO}^-] \nonumber \\&=[\mathrm{XOAc}]+[\mathrm{AcO}^{-}]\left( 1+{\frac{[\mathrm{H}^{+}]}{K_{AcOH}}}\right) \end{aligned}$$

and A as

13 $$\begin{aligned}{}[\mathrm{H}_{2}\mathrm{A}]_o&= [\mathrm{HA^{-}}]+ [\mathrm{A}^{2-}] = [\mathrm{A}^{2-}]\left( 1+\frac{[\mathrm{H}^{+}]}{K_{\rm {a}}}\right) \end{aligned}$$

with use of Eqs. (5) and (8), respectively. To continue, the charge neutralization conservation equation is invoked, i.e.

14 $$\begin{aligned}{}[\mathrm{AcO}^{-}]+[\mathrm{OH}^{-}]+[\mathrm{HA}^{-}]+2[\mathrm{A}^{2-}] = [\mathrm{H}^{+}]+ 2[\mathrm{M}^{2+}] \end{aligned}$$

which can be expressed as

15 $$\begin{aligned} \left( \frac{[\mathrm{XOAc}]_o-[\mathrm{XOAc}]}{\left( 1+\frac{[\mathrm{H}^{+}]}{K_{AcOH}}\right) }\right) &+\frac{K_{\rm {w}}}{[\mathrm{H}^{+}]}+[\mathrm{H}_{2}\mathrm{A}]_0\left( \frac{[\mathrm{H}^{+}]+2K_{\rm {a}}}{[\mathrm{H}^{+}]+K_{\rm {a}}} \right) \nonumber \\=[\mathrm{H}^{+}] &+ 2\left( \frac{[\mathrm{MO}]_0-[\mathrm{MO}]}{\left( 1+\frac{\mathrm{K}_\mathrm{w}^{2}}{K_{\rm {m}}[\mathrm{H}^{+}]^{2}}\right) }\right) \end{aligned}$$

through use of Eqs. (11)–(13). This equation indicates that the proton concentration in the solution is governed by charge neutralization and is related to moles of acetic acid formed (first term on LHS of equation), the amount of ash neutralized (second term on RHS of equation), three different equilibria found in solution $(K_{\rm {m}},\,K_{\rm {a}},\, K_{\rm{AcOH}})$, and the amount of acid initially added (${[\mathrm{H}_{2}\mathrm{A}]_0}$). To complete this description, we use the rate expressions given in Eqs. (4) and (9)

16 $$\begin{aligned} \frac{\rm {d}}{{\rm {d}}t}[\mathrm{XOAc}]= & {} -k_1[\mathrm{XOAc}][\mathrm{H}^{+}] \qquad [\mathrm{XOAc}(0)] = [\mathrm{XOAc}]_{0} \end{aligned}$$

17 $$\begin{aligned} \frac{\rm{d}}{{\rm {d}}t}[\mathrm{MO}]= & {} -k_{2}[\mathrm{MO}][\mathrm{H}^{+}] \qquad [\mathrm{MO}(0)] = [\mathrm{MO}]_{0}, \end{aligned}$$

where the subscript o represents the initial concentration of the species. Eqs. (15–17) represent the toy model to describe the proton concentration during reaction. The utility of this set of equations was tested in three separate experiments:

1. At long reaction times where the reactions with XOAc and MO are nearly complete.
2. With the reaction occurring at room temperature to examine the proposed ash neutralization scheme.
3. At typical reaction temperatures found for prehydrolysis as a function of initial pH.

## Methods

Bamboo chips, provided by the Lee & Man Paper Manufacturing Ltd., were stored at 4 °C until used for experimentation. The chips were air dried for approximately 24 h and re-chipped using a Wiley mill (Thomas Scientific, NJ, USA) and screened with a 45–16–9.5 mm stacked sieve system. Chips retained on the 9.5 mm pan were designated as accepts for experimentation. The accepts were washed twice (6 and 4 min, respectively) with distilled water at a liquid to wood (based on the oven dry weight of wood) ratio of L:W = 20:1 using a laboratory mixer. The washed chips were air dried for approximately 24 h and stored at 4 °C until used.

Before starting, the chemical composition of the accept chips were analyzed following National Renewable Energy Laboratory (NREL) standard protocols [36]; see Table 1 for a summary of the results. Briefly, the chips were air-dried and ground to pass through 40-mesh using a Wiley mill. The powdered samples were then digested by a two-step H_2_SO_4_ hydrolysis protocol. For polysaccharide analysis, acid hydrolysates (liquid samples) were recovered by filtration through medium-porosity filtering crucibles (Fisher Scientific Co., ON, Canada), and an internal standard, fucose, added. These samples were re-filtered using 0.2 μm syringe filters (Chromatographic Specialties, Inc. ON, Canada) for HPLC. A Dionex ICS 5000+ HPLC system fitted with an AS-AP autosampler was used to separate the monomeric sugars in the samples at 45 °C, against sugar standards, on a Dionex CarboPac SA10 analytical column. 1 mM NaOH at 1 mL/min flow was the mobile phase, and the sugars were quantified using electrochemical detection and Chromeleon software (Thermo Fisher Scientific, MA, USA). High-purity monomeric sugar standards, arabinose, galactose, glucose, xylose and mannose were purchased from Sigma-Aldrich (ON, Canada).

A portion of the filtrate recovered after the two-step acid hydrolysis was analyzed for acid soluble lignin following [37]. Acid insoluble lignin was determined gravimetrically according to Sluiter et al. [36]. TAPPI test method T211 om-02 was followed to determine the total ash content. Detailed analysis of the metal composition of ash was done using inductively coupled plasma time of flight mass spectrometry (ICP-TOFMS) [38]. The α- and β-cellulose content of bamboo were determined according to TAPPI test method T203 om-09.

Four separate studies were conducted in this work, as summarized in Table 2. In all cases, bamboo chips and water were mixed at defined liquor to wood ratios (L:W, see Table 2) and placed in a 300-mL stainless steel reactor. The total mass of the chips and water for all L:W ratios was kept constant at 217 g; this slurry filled about 80 % of the available volume of the reactor. The purpose of the first study (series 1–10) was to characterize the reactor temperature response over time. The reactor was immersed in an oil bath set at a defined temperature, $T_{\rm {b}}$. The temperature of the mixture was continually monitored with two thermocouples mounted in the middle of the reactor, on the central plane but at two different radial positions. Upon completion of a run, the reactor was cooled by immersion in an ice-water bath.

In the second study (series 11 and 12), conducted to investigate the equilibrium proton concentration after a long period of time, the pH of bamboo chips–liquid mixture having a liquor-to-wood ratio of L:W = 6.5:1 (wt/wt) was measured as a function of initial acid content after a minimum of 6 h (in some cases 10 h). In the third study, this was repeated but conducted at room temperature for more than 10 h (series 13–16). In the fourth study (series 17–21), the time evolution of the proton concentration was measured as a function of time, temperature and initial acid addition. In this case, the L:W = 6.5:1 (wt/wt).

## Results and discussion

**Fig. 3:**
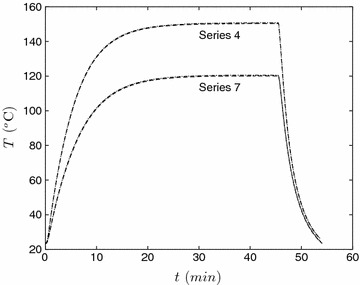
Temperature evolution for two representative cases. Both thermocouple signals are presented and replicate runs are shown but the difference between them is not perceptible on this scale. The thermocouples are place at the same elevation in the reactor but at two different radial positions. The experimental conditions for each series are given in Table 2

Before proceeding to the main findings, it is instructive to first examine the temperature profile in the reactor after immersion in the oil bath. For each experimental condition, the temperature of the reaction mixture (chips and liquid phases) was recorded using two temperature transducers located at different radial positions in the reactor. In all conditions (series 1–21) there was no significant difference between the two transducer signals, and the reactor seemingly behaved as if there were no spatial gradients in the system, i.e. it was at a uniform temperature. Two results representative of all runs are shown in Fig. 3. The trend with all data sets was that the heat-up period was 15~20 min, i.e. the heat-up rate was essentially the same. The cool-down rate was approximately ~25 °C.

It curious that there are no strong radial temperature gradients in the system. This result is evident in both the pure water case (series 1–2) and cases with L:W ratios as low as 6.5:1 (series 3–6). We offer two speculative arguments to explain this. In the first case, we argue that the thermal mass of the steel reactor, i.e. the product of its mass and heat capacity, to be significantly larger than the reactants. As a result, the temperature response of the reactants is dictated by the heating or cooling of the reactor. The second argument is somewhat more delicate. It is also possible that convection occurs due to difference in density of the fluid near the outer wall in comparison to the bulk. Convective currents in the reactor would tend to diminish the radial gradients.

**Fig. 4:**
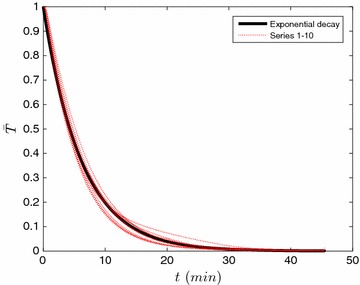
The temperature evolution during the heat up period for series 1–10. The results have been scaled using the form advanced in Eq. (18) with h/c set to be 0.15/min

With the notion of uniform spatial temperature gradients, we pose a second toy model to understand the temperature evolution throughout the reaction. We propose the temperature profile follows an equation of the form

18 $$\begin{aligned} c\frac{{\rm {d}}T}{{\rm{d}}t} = h(T_b-T) \Rightarrow \bar{T}= \frac{T_{\rm {b}}-T}{T_{\rm {b}}-T_0} = \exp \left(-\frac{h}{c}t\right) \end{aligned}$$

where *c* is the product of the effective mass and heat capacity of the reactor and reactants; and *h* is the overall heat transfer coefficient. The utility of this equation is tested by plotting series 1–10, shown in Table 2, in Fig. 4 using the scalings indicated in Eq. 18. What is evident in this figure is that the system displays nearly exponential behavior as the experimental data (the red dotted lines) somewhat follow Eq. 18, shown as the thick black line. However, we were unable to achieve a similar scaling during the cool-down period.

**Table 2:** A summary of the experimental conditions tested

Experiment	Series	L:W (wt / wt)	${[H{^+}]_o}$ (pH)	*T*_b_ (°C)	*t* (min)
Temp. measurement	1	water only	7.1	120	0 < *t *<45
	2	water only	7.1	150	0 < *t* <45
	3	6.5:1	7.1	140	0 < *t* <45
	4	6.5:1	7.1	150	0 < *t* <45
	5	6.5:1	7.1	160	0 < *t* <45
	6	6.5:1	7.1	180	0 < *t* <45
	7	8:1	7.1	120	0 < *t* <45
	8	8:1	7.1	150	0 < *t* <45
	9	10:1	7.1	120	0 < *t* <45
	10	10:1	7.1	150	0 < *t* <45
Long-time behavior	11	6.5:1	1.3–6.8	160	*t* >315
	12	6.5:1	1.5–7.1	180	*t* >315
Room temperature	13	6.5:1	1.5	23	0 < *t* < 1155
	14	6.5:1	2.9	23	0 < *t* <1155
	15	6.5:1	5.0	23	0 < *t* <1155
	16	6.5:1	6.0	23	0 < *t* <1155
Elevated temperature	17	6.5:1	1.7	160	0 < *t* <360
	18	6.5:1	1.5	160	0 < *t* <390
	19	6.5:1	7.2	160	0 < *t* <390
	20	6.5:1	7.2	180	0 < *t *<390
	21	6.5:1	3.5	160	0 < *t* <360

In series 1 through 10, the temperature was sampled at a frequency of 1 *Hz*. In series 11–12 a total of 18 samples were measured at times ranging from 315 to 390 min. In series 13–14, four different initial pHs were tested and approximately 11 samples, obtained at different times, were measured. In this case $T_b$ is defined as the oil bath temperature. L:W and t refer to liquid to wood ratio and time

At this point, we begin to explore the utility of the toy model. The first aspect of the model that we will explore is the long-time behavior and examine if a steady-state proton concentration is possible. Experimentally, the pH was measured at long time by simply allowing the reaction to proceed for at least 315 min at an elevated temperature. From the toy model, we see that a steady-state concentration for [H^+^] exists and can only be achieved when both the deaceytlyation and neutralization reactions are complete, i.e.

19 $$\begin{aligned} \frac{{\rm {d}} [\mathrm{XOAc}]}{{\rm {d}}t}=\frac{{\rm {d}} [\mathrm{MO}]}{{\rm {d}}t}=0, \quad \text {thus,} \quad [\mathrm{XOAc}]=[\mathrm{MO}] = 0. \end{aligned}$$

**Fig. 5:**
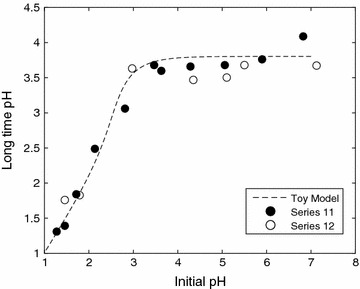
The effect of initial pH on the steady-state pH measured after long-time prehydrolysis. The dashed line represents the toy model with the equilibrium constant given in the text previously. The value of *K*_m_ is not stated in the text and is taken to be small. For practical purposes $K_{\rm {m}}=10^{-17}\,{\rm M}$ for this calculation. The two remaining parameters are set to be ${[\mathrm{{XOAc}}]}_o=0.025\,{\rm M}$ and ${[\mathrm{{MO}}]}_o=0.001\,{\rm M}$ and were determined through regression. Series 11 represents the reaction at 160 °C and series 12 at 180 °C

Indeed, at steady state the proton concentration may be estimated directly from Eq. 15, i.e.

20 $$\begin{aligned} \frac{[\mathrm{XOAc}]_{0}}{\left( 1+\frac{[\mathrm{H}^{+}]}{K_{\rm {AcOH}}}\right) }+\frac{K_{\rm {w}}}{[\mathrm{H}^{+}]}+[\mathrm{H}_{2}\mathrm{A}]_{o}&\left( \frac{[\mathrm{H}^{+}]+2K_{\rm {a}}}{[\mathrm{H}^{+}]+K_{\rm {a}}} \right) \nonumber \\&= [\mathrm{H}^{+}]+ 2\frac{[\mathrm{MO}]_{0}}{\left( 1+\frac{{K}_{\rm {w}}^{2}}{K_{\rm {m}}[\mathrm{H}^{+}]^{2}}\right) } \end{aligned}$$

which is a sixth-order polynomial in [H${^+}$]. The steady-state concentration is given by the roots of this polynomial and the behavior of this function is given in Fig. 5. This equation was solved for [H${^+}$] in MATLAB using the built-in root finding procedure. Superimposed on this is the experimental data given as series 11 and 12. Two observations are clearly evident. The first observation that can be made is that we find a remarkably similar trend with the toy model. The second observation is that there are two distinct regions. Under autohydrolysis conditions, i.e the right-hand potion of the graph, the steady-state (or long time) pH is independent of the initial pH. Here, the steady-state pH is governed by the weak-acid equilibrium and by ash neutralization or buffering. With increasing levels of added acid, we find that the long-time pH approximately equals the initial pH. This is shown on the left-hand portion of Fig. 5.

These results support the kinetic modeling for xylan removal under dilute-acid conditions. As discussed in the introduction, a number of authors have assigned the proton concentration to be constant and equal to its initial value (see [11, 12, 24, 31] for example). However, under autohydrolysis conditions, this does not occur. There is a vast difference between the initial and the steady-state pH of the system.

**Fig. 6:**
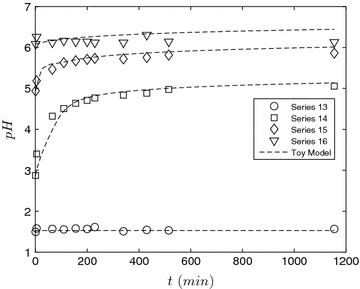
Examination of the pH when the reaction proceeds at room temperature. To evaluate the toy model, the same values for the constants given in the caption of the previous figure are used. In addition, $k_2 = 10 \, {\rm M}^{-1}{\rm min}^{-1}$ as determined by regression

We continue the discussion of the toy model and examine a second limiting case when the reaction proceeds at room temperature, see Fig. 6. Here, four cases were examined in which the amount of acid initially added was varied. At room temperature, it can be assumed that the deacetylation reaction proceeds at a much slower rate in comparison to the ash neutralization scheme. Under this assumption, the toy model reduces to

21 $$\begin{aligned} \frac{\rm {d}}{{\rm {d}}t}[\mathrm{MO}]&= -k_2[\mathrm{MO}][\mathrm{H}^{+}] \end{aligned}$$

22 $$\begin{aligned} \frac{K_{\rm {w}}}{[\mathrm{H}^{+}]}+[\mathrm{H}_{2}\mathrm{A}]_{0}\left( \frac{[\mathrm{H}^{+}]+2K_{\rm {a}}}{[\mathrm{H}^{+}]+K_{\rm {a}}} \right)&= [\mathrm{H}^{+}]+ 2\left( \frac{[\mathrm{MO}]_{0}-[\mathrm{MO}]}{\left( 1+\frac{{K}_\mathrm{w}^{2}}{K_{\rm {m}}[\mathrm{H}^{+}]^{2}}\right) }\right) \end{aligned}$$

which has been solved numerically in MATLAB using a Runge–Kutta scheme (ODE23s) coupled with a root-finding procedure for the proton concentration. The equations are solved simultaneously. As shown in Fig. 6, at low initial pH (series 13), pH is constant as the concentration of added acid is in excess of the neutralization potential of the ash. With decreasing initial added acid (series 14–15), the neutralization reaction proceeds until all the ash is reacted. With series 16, no added acid, the pH varies weakly with time. We interpret this result through the toy model, and advance the argument that the neutralization reaction proceeds but the kinetics are extremely slow due to the low proton concentration.

**Fig. 7:**
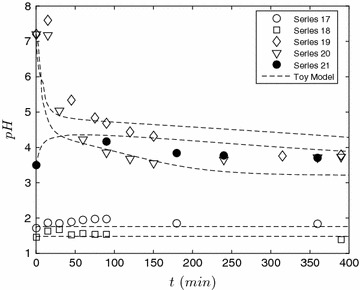
A evolution of pH under prehydrolysis conditions. To help understand this, we related the lowest initial pH experiments to the added acid: series 17 0.25 % (wt/wt) and series 18 0.5 % (wt/wt)

We now move to perhaps the main findings of this work and examine the evolution of pH during prehydrolysis treatment. In our final set of experiments, we examine the evolution of the proton concentration at elevated temperatures. Here we must include the effect of deacetylation and as a result, the full toy model must be solved numerically using MATLAB. We treat Eqs. 16–17 as a system of equations and solve this initial value problem in conjunction with a root-finding procedure to estimate [H${^+}$] from Eq. 15. The results are shown in Fig. 7. Again at low initial pH, proton concentration varies weakly with time (cf series 17 and 18) and remains essentially at its initial value. This was the expected result as demonstrated earlier through steady-state analysis under excess acid conditions, the pH should remain essentially constant. Below this limit complex behavior is observed. Under autohydrolysis conditions (series 20 and 21), there is a rapid initial drop in pH followed by a diminished rate at longer times. However, the most curious result is given by series 21 where non-monotonic behavior, i.e. the pH initially rises and then falls, is observed. We base our interpretation on the toy model which indicates that the neutralization reaction is initially proceeding faster than deacetylation. At longer times, the ash is completely reacted and the pH diminishes from deacetylation.

These results can now be used to interpret the form of the rate constant used for xylan removal. As seen in Eq. 3, the rate constant under dilute acid conditions is related to the initial pH. This is quite reasonable as we have shown that the pH should be essentially constant during the course of the reaction. We, however, cannot make any comment on the value of *n* in this equation. Below this limit, the behavior of [H${^+}$] is very complex. Simple linear functions may indeed apply for a particular systems of interest. However, this cannot be generalized as the pH response depends strongly on the rate of ash neutralization in comparison to the rate of deacetylation.

## Summary and conclusions

In this work, the evolution of the proton concentration was examined during the hydrolysis of bamboo chips. At issue was the seemingly disparate model descriptions in the literature which treat dilute acid differently than autohydrolysis conditions. We have attempted to address this issue by posing a “toy model” in which we have included a number of chemical components to help describe the reaction. We advance that the proton concentration is governed by a charge neutrality in the solution and influenced by the:

1. weak-acid equilibrium formed from the deacetylation of the acetyl group from the xylan,
2. equilibrium created by water dissociation,
3. ash neutralization and the associated equilibrium in solution,
4. added acid.

There are a number of outcomes from the toy model which have been tested experimentally. The first, and perhaps most significant outcome, is that the toy model predicts the existence of a steady-state solution. The steady-state value is dictated by the equilibrium constants, and the initial added acid and acetyl group levels. The model qualitatively follows the trend given by experiment. It is difficult to perform a quantitative comparison as auxiliary relationships, such as the variation of equilibrium constant with temperature, are not known. The model was tested at room temperature to examine the changes in pH when ash neutralization is the dominant mechanism. Under these conditions we find, surprisingly, that the pH remains essentially constant both at low initial pH and under autohydrolysis conditions. Acid is likely in excess of the neutralization potential of the ash, in the former case, and the kinetics of neutralization become exceedingly small in the latter case due to the low proton concentration. Finally, when the hydrolysis reaction proceeded at elevated temperatures, we found one case of non-monotonic behavior in which the pH initially increased, and then decreased at longer times. This is attributed to the difference in rates between the neutralization and deacetylation reactions.

As described in the introduction the evolution of the proton concentration during prehydrolysis is poorly modeled using empirical functions [Eq. (5)] which are not rooted in a proper chemical reaction scheme. With our toy model, we propose a chemical reaction pathway that satisfactorily describes experimentally determined proton concentration under both autohydrolysis and dilute-acid hydrolysis conditions. Accurate modeling of the proton concentration would significantly improve the existing kinetic models of hemicellulose hydrolysis and facilitate more efficient process optimization and scale-up.

## Abbreviations

Ac: acetyl group H_3_C–C(=O)−
Ar: arabinose
a: empirical constant in Eq. (5)
b: empirical constant in Eq. (5)
c: product of the effective mass and heat capacity of the reactor and reactants
*E*_ai_: activation energy of reaction i
H_2_A: acid species
*h*: overall heat transfer coefficient
*K*_i_: equilibrium constant at room temperature for reaction* i*
*k*_i_: rate constant for reaction *i*
*k*_oi_: pre-exponential factor for rate constant *k*_i_
LHS: left-hand side
L:W: liquid to wood ratio
MO: oxide of ash species M with a valence state of 2^+^
*n*: empirical constant describing power law dependence of *k*_i_ on proton concentration
RHS: right-hand side
*T*: temperature
*T*_b_: oil bath temperature
*t*: time
*X*: xylose
